# Autonomous sensory Meridian response as a physically felt signature of positive and negative emotions

**DOI:** 10.3389/fpsyg.2024.1183996

**Published:** 2024-03-01

**Authors:** Wai Lam Leung, Daniela M. Romano

**Affiliations:** ^1^Department of Psychology and Language Sciences, University College London, London, United Kingdom; ^2^Department of Psychology, Institute of Psychiatry, Psychology and Neuroscience, King’s College, London, United Kingdom; ^3^Department of Information Studies, University College London, London, United Kingdom; ^4^Institute of Artificial Intelligence, De Montfort University, Leicester, United Kingdom

**Keywords:** autonomous sensory meridian response (ASMR), frisson, relaxation, fear, emotional valence, emotional arousal, neutral response, mood

## Abstract

**Introduction:**

Current research on Autonomous Sensory Meridian Response (ASMR) assumes that ASMR is always accompanied by contentment, and it is distinct from frisson due to positive emotions. Thus, research investigations tend to limit their scope to solely focusing on the sensation of relaxation that ASMR induces. This study explores whether it is possible to have a different emotional experience and still perceive ASMR, testing the theory of ASMR as an amplifier of pre-existing emotion instead of a determination of positive affect.

**Methods:**

The emotional arousal and valence, and mood changes of 180 ASMR-capable and incapable individuals were analysed using questionnaires after altering the affective interpretation associated with auditory ASMR (tapping) with visual priming to examine whether the primed emotion (fearful, relaxing, or neutral) could be amplified.

**Results:**

It was found that an ASMR response occurred in all priming conditions, including the fear priming group. No significant difference was found in the emotional outcome or mood of the neutral and relaxing priming groups. Upon comparison with ASMR-incapable individuals, both the relaxing and neutral priming groups demonstrated the same affect, but greater potent for ASMR-capable. Individuals who appraised ASMR after visual fear priming demonstrated a significant decrease in positive emotional valence and increased arousal.

**Conclusion:**

The findings suggest that ASMR occurs in both positive and negative emotional situations, suppressing contentment induction if ASMR stimuli are interpreted negatively and amplifying contentment when interpreted positively. While more research is needed, the results highlight that ASMR and frisson might describe the same phenomenon, both a physically felt signature of emotion. Therapeutic usage of ASMR should carefully select appropriate stimuli that emphasise contentment to avoid potential health risks associated with negative emotions until a further understanding of ASMR’s affective parameters has been established.

## 1 Introduction

Autonomous Sensory Meridian Response (ASMR) is a perceptual response that begins from the scalp or spine and spreads across the body upon exposure to specific external triggers. Similar to frisson, a bodily sensation that involves a pleasurable chill that originates similarly around the head, neck, and spine area (Colver and El-Alayli, 2016), ASMR has been described as a form of “low-grade euphoria” due to its accompaniment with feelings of relaxation (Barratt and Davis, 2015; Fredborg et al., 2017; Poerio et al., 2018). Most research suggests that ASMR triggers vary from person to person, where triggers can be auditory, visual, tactile, or an amalgamation of them all (Barratt et al., 2017; Fredborg et al., 2017). ASMR stimuli are highly accessible through the internet; its accessibility being a key factor in its popularity over recent years.

The definition of ASMR has become ambiguous or overly subjective, promoting difficulties in distinguishing the differences between ASMR and other similar sensations, i.e., frisson or synaesthesia (Poerio et al., 2022). Where for example, synaesthesia, the involuntary perception that crosses over between senses (e.g., one is able to hear sounds and see colors), is common among those perceiving ASMR (Poerio et al., 2022). Descriptions of the ASMR sensation often mirror ones used to describe frisson (Panksepp, 1995; Blood and Zatorre, 2001). The similarity between the descriptions of the two phenomena has raised multiple stances within the research community. Some have argued that ASMR is a gentler subclass of frisson (Fairyington, 2014), whereas others would claim ASMR is unique to frisson and an independent sensation (Fredborg et al., 2017). Although the two are nearly indistinguishable at a descriptive level, empirical evidence has suggested that the differences between them predominantly lay upon the triggers that initiate them. For instance, frisson is mostly reported during music-listening and esthetic experiences compared to ASMR, which is more commonly experienced during mundane activities (e.g., physical examinations; Ahuja and Ahuja, 2019).

Due to the difficulties of distinguishing ASMR definition from frisson aside from ASMR-inducing contentment, research may have neglected contradicting claims related to non-relaxing ASMR experiences to maintain a forced distinction between the two. Although some empirical evidence does suggest that ASMR is associated with relaxation from psychometrics and physiological measurements, investigations tend to limit their scope to solely focus on the sensation of relaxation to avoid variating from the original non-scientific description of ASMR raised from the internet (see Barratt and Davis, 2015; Fredborg et al., 2017).

However, Kovacevich and Huron (2019) conducted a content analysis of the 30 most popular ASMR videos to examine the required features of ASMR. ASMR videos used less music, were quieter, and tended to use private settings as opposed to public settings when compared to control videos that were not ASMR-like in nature. This distinguishes ASMR from frisson, which is often reported to be under situations involving the presence of music and can occur during a loud listening session (Fredborg et al., 2017). However, both ASMR and control video ratings yielded a low Kappa statistic of 0.47, implying only moderate agreement within interrater reliability. Nevertheless, these findings were triangulated with self-report data, which found that using similar triggers induced ASMR in participants across different research studies (Barratt et al., 2017). This, therefore, shows the importance of distinguishing ASMR-like experiences from experiences of frisson. However, there are some similarities between ASMR and frisson, particularly in their physiological symptoms. For instance, pleasurable pilomotor activation (e.g., goosebumps) is shared across both sensations (Blood and Zatorre, 2001), and both would induce an increase in galvanic skin response (Guhn et al., 2007; Poerio et al., 2018). Thus, it is difficult to distinguish whether the bodily sensation is a result of ASMR if the context of triggers is insufficient or ambiguous. This ambiguity in identifying ASMR is even more problematic when research has shown that the triggers vary from person to person (Fredborg et al., 2017). Therefore, it is inherently difficult to distinguish the physiological sensations between ASMR and frisson.

Many have argued that the most critical component of distinguishing ASMR from other bodily sensations is the emotional outcome (Barratt and Davis, 2015; Del Campo and Kehle, 2016; Fredborg et al., 2017). As previously mentioned, ASMR is frequently associated with contentment and improvement in subjective wellbeing (Bishop et al., 2004; Barratt and Davis, 2015). For instance, Lohaus et al. (2023) demonstrated that ASMR sensations triggered simply by viewing ASMR videos are related to higher state relaxation and positive affect scores. This nature of the phenomenon became the primary reason why ASMR gained popularity because of its positive influence on emotion without complicated procedures. This led to comparisons between ASMR and mindfulness, a meditation method that involves distributing attention toward moment-by-moment experiences to repel negative emotions (Bishop et al., 2004). The premise of mindfulness is to gain awareness of the present and reduce reactiveness toward surrounding events or thoughts that may negatively influence our feelings (Lutz et al., 2008). The similarities between mindfulness and ASMR are not limited to the outcome but also procedural elements since recent research has discovered intertwined processes such as attentional control and openness to sensations (Fredborg et al., 2018). Thus, ASMR has caught some attention in the clinical field due to its potential to be an innovative medium for stress management and overall mental wellbeing (e.g., Cash et al., 2018).

However, esthetic chills such as frisson are often associated with excitement and physiological arousal (Grewe et al., 2009; Del Campo and Kehle, 2016). Supported by physiological measurements, the cardiac reaction upon experiencing the two sensations demonstrated a polarizing effect. While esthetic chills increase respiration rate and respiratory depth (Benedek and Kaernbach, 2011), ASMR reduces heart rate (Poerio et al., 2018), which can indicate relaxation (Pittig et al., 2013) since excitement and arousal are often associated with an increase in heart rate (Wulfert et al., 2005). However, Poerio et al. (2018) asked participants about their experiences with ASMR, and they reported increased levels of subjective excitement despite the reduction in heart rate. Regardless, the experience overall is still associated with contentment, which is not a component of frisson. Although distinguishing one phenomenon from another is undoubtedly an informative milestone in ASMR research, findings aligned with people’s expectations have generated unnecessary strictness in bodily sensation classification. This strictness has manifested within internet communities—deeming reported anomalies within ASMR experience as preposterous. Claims regarding individuals experiencing non-relaxing ASMR are often disregarded due to the strict definition that ASMR must be accompanied by contentment. The findings of ASMR research can be misinterpreted by the layperson as findings not only alter the public’s beliefs of ASMR but also the productivity of scientific research. ASMR research relies on content produced by the public, which may be reduced if people deliberately cancel meaningful debates on the topic. Astonishingly, these contradicting claims have remained resilient and begun to surface.

Non-relaxing ASMR has been reported on the internet for an extensive duration but was never acknowledged by the scientific community. For instance, a researcher of this project found that there are a few Reddit forum posts regarding ASMR as accompanied by fear responses. However, these experiences have caught little attention because they differ so strongly from our current understanding of the ASMR paradigm, and therefore, alternative experiences of ASMR have been neglected by public interest. One of these discussions depicted a panic episode when walking alone in the woods at night that was accompanied by ASMR (see I Started to Notice, 2016). Moreover, some YouTube videos have been suggested to be ASMR inducing even though they have unusual triggers; for example, videos of individuals walking into various locations (e.g., Aokigahara “Suicide” Forest & Cemetery; Rambalac, 2018). Although these videos may typically be associated with fear, they were suggested to be ASMR-inducing by both the uploader and the viewers, albeit with some uncertainty due to the divergence from the typical ASMR paradigm.

Another type of non-relaxing ASMR that has been reported is the association of ASMR with feelings of disgust. This experience is far more common than fear-related ASMR and is the reason why many individuals do not enjoy listening to ASMR-related stimuli. Most would argue that the reason for expressing disgust toward ASMR is related to misophonia, which refers to the hatred of sounds (Kovacevich and Huron, 2018). The typical trigger for misophonia is human-generated noises such as eating and breathing (Diamond-Flower, 2018; Rouw and Erfanian, 2018). Upon exposure to ASMR, an individual with misophonia would feel intense disgust, anger, or anxiety as an aversive response to avoid further exposure to the sound source (Wu et al., 2014). This experience of disgust is most prevalent in ASMR eating videos, where viewers either find it incredibly relaxing or disgusting (see Diamond-Flower, 2018; ima-lick-u, 2018).

Smith and Snider (2019) provided multiple references to ASMR artist’s emphasis that ASMR media is intended for relaxation, stress, and anxiety management (Andersen, 2015), and there is undoubtedly a strong influence within the ASMR community to enforce the association between relaxation and ASMR. While most ASMR artists have enforced that the tingling sensation is a “relaxation response” (Andersen, 2015), no empirical findings can be confident that the response itself is relaxing by default. Specifically, based on the literature, we cannot distinguish whether ASMR is an esthetic chill-like experience with a relaxing nature or it is just a frisson generated by perceptual triggers that occur independently from emotions and acts as a physically felt signature of emotion, where contentment is just a by-product of interpretation since Smejka and Wiggs (2022) suggested that ASMR videos induce relaxation regardless of whether one can experience ASMR. However, the potency of relaxation has been shown to be more pronounced in individuals who can experience ASMR, which may indicate that ASMR sensations serve to amplify pre-existing emotions induced by the affective interpretation of the positive ASMR stimuli.

Thus, the assumption of ASMR sensation is not to determine emotion, but rather, to amplify pre-existing emotions. Perhaps one could attempt to manipulate one’s affective positive interpretation of ASMR stimuli and cause ASMR sensation to amplify alternative emotions. Moreover, if such a hypothesis is correct, then there is a need to avoid narrowing our scope in believing that ASMR is always accompanied by relaxation and that there might be a need for re-evaluation in previous literature since most influential research has acquired samples from ASMR-related communities, which can be biased toward interpreting ASMR stimuli as relaxing. Anticipating ASMR as a relaxation response that produces biased positive affect findings is a self-fulfilling prophecy. More importantly, as mentioned earlier regarding the abundance of attention on ASMR being a potential therapeutic tool in the professional scene, if ASMR is indeed a signature or an overwhelming pre-existing emotion, then a stricter framework should be proposed when using ASMR in a therapeutic capacity, as it may be harmful when interpreted negatively. Thus, we intend to test whether it is possible to produce alternative emotional outcomes from ASMR by manipulating one’s affective interpretation of the stimuli.

To test whether ASMR is a physically felt signature of an overwhelming emotion, researchers can prime the interpretation of the context of ASMR triggers to instigate a specific emotion with visual information (i.e., a video) beforehand. The visual information alone should induce an emotion that ASMR will later physically amplify (e.g., finger-tapping noises may be interpreted as blood dripping if primed by gory visuals beforehand, which may induce and amplify fear upon ASMR exposure). The priming material should be congruent to the ASMR trigger, meaning visual information must relate to the auditory ASMR trigger. Otherwise, the discrepancy between visual and auditory information may render priming ineffective.

The current experiment focuses on priming auditory ASMR triggers, specifically finger tapping, to test whether a negative emotion (e.g., fear) can be induced and physically amplified during the ASMR experience. Finger-tapping triggers were chosen due to their popularity and implementation in literature (Barratt et al., 2017; Fredborg et al., 2017). Most importantly, finger tapping is suitable for priming since it is more contextually neutral than triggers such as whispering, which is commonly misinterpreted sexually (Smith and Snider, 2019), or chewing, which may be considered ill-mannered and invoke disgust, for better control of manipulation across conditions. Furthermore, we chose to investigate fear due to its direct polarizing characteristics to contentment for better contrast in findings (McGinn and Kelly, 2018; Gu et al., 2019).

Overall, in this experiment, there will be three priming conditions: relaxation, fear, and neutral emotion (control condition) to evaluate the physical emotion amplification theory. Relaxation conditions will be primed using the conventional ASMR visuals, fear conditions using fear-related visuals, and control using emotionally neutral visuals. Concerning the control group, we hypothesize that the effect of ASMR on emotional amplification may be less potent than individuals primed with contentment-related visuals since previous studies have demonstrated that misophonia is influenced by the amount of contextual information given (see Edelstein et al., 2020). Thus, we hypothesized that control groups would demonstrate contentment induction due to the content impression of ASMR stimuli caused by media, as mentioned earlier, but not as strongly as the relaxation priming condition. Furthermore, individuals who do not normally have ASMR response toward the intended stimuli (i.e., ASMR-incapable group) will be tested for the three conditions as well to provide additional data for comparison with their capable counterparts to further affirm our theory. Thus, we hypothesize that ASMR-incapable individuals of each priming group will exhibit emotional valence and arousal of the same direction in relation to their ASMR-capable counterpart with lessened intensity as the absence of ASMR sensation in the ASMR-incapable group would disable amplification of the primed emotion.

## 2 Materials and methods

### 2.1 Participants

A total of 181 participants were recruited from the ASMR Reddit subforums^1^ and through word-of-mouth. Reddit is a forum bringing together an English-speaking community with rules and terms and conditions written in English. Thus, participants’ English comprehension abilities were assumed to be adequate for the experiment. Participants were entered in a prize draw to win a £10 Amazon voucher for their participation. One participant was excluded due to inappropriate responses during participation, which left 180 valid participants (104 male, 68 female, and 8 others). The ages of the participants ranged from 18 to 55 years (*M* = 26.47, *SD* = 6.82). The participant’s origins can be divided into three main groups: 53 were European (29.4%), 79 were Americans (43.9%), and 48 were from other countries (26.7%; e.g., South East Asians, Eastern Asians, Africans, and South Americans). An a priori power analysis was conducted using G*Power version 3.1.9.7 (Faul et al., 2007) to determine the minimum samples size required for 2 × 3 factorial MANOVA at medium effect size ($f^{2}$ = 0.15; Cohen, 1988) and an alpha of 0.05 was 60 samples to achieve a power of 0.80.

**Figure 1:**
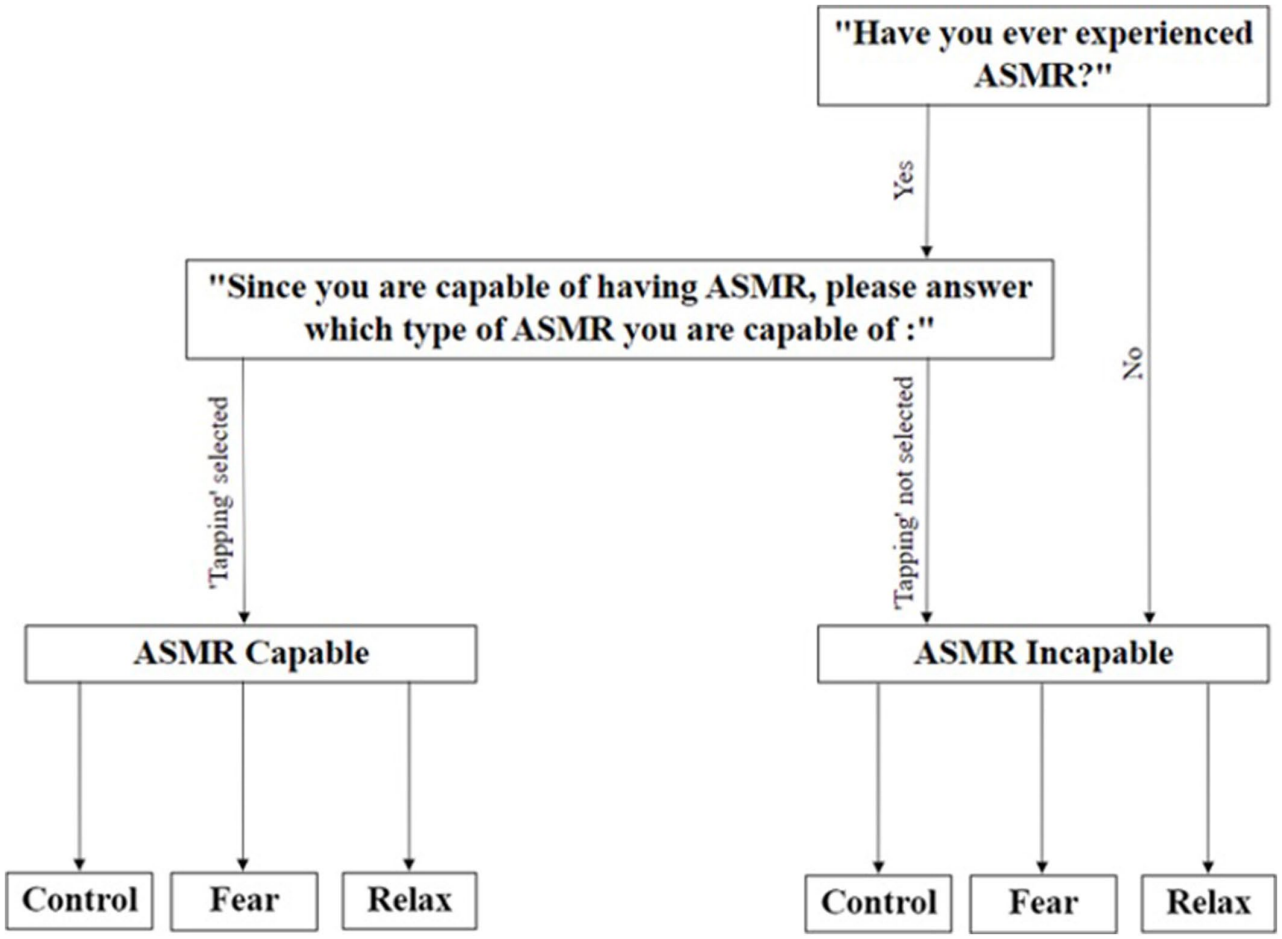
New SAM ASMR intensity scale (1 = No ASMR, 9 = Extreme ASMR intensity).

Upon participation in the study, participants were labeled according to their ASMR ability (ASMR-capable or incapable) and the type of ASMR stimuli they were triggered by, which were assessed through the initial ASMR checklist (see Figure 1). Individuals who had not experienced ASMR and participants who were not triggered by auditory “tapping” stimuli were placed in the group labeled “ASMR Incapable” for the purpose of the experiment. Participants triggered by tapping were allocated to the “ASMR Capable” group (see Figure 1). The second criterion is crucial given that Fredborg et al. (2017) suggested that trigger type might vary among ASMR-capable individuals; this allows us to identify which participants can be triggered by the experimental stimuli and use ASMR-incapable individuals as a baseline to examine the effect of ASMR.

After assessing ASMR capability, participants are randomly allocated to either control, fear, or relaxation priming conditions (see Figure 1). Informed consent was obtained from all participants who took part in the study. All data were anonymized, and participants could withdraw at any point in the study. Ethical approval for this research was granted by the Department of Information Study Ethics Chair, University College London.

### 2.2 Measures

#### 2.2.1 Experiment program

The study was created using an online experiment builder program Gorilla™. The experimental program can be accessed with a desktop, laptop, computer, or tablet. Participants were allowed to activate the program through any platform, but computers were recommended due to better technical optimization.

#### 2.2.2 ASMR audio

Five relaxing tapping ASMR videos were retrieved from YouTube based on view count since the popularity could represent audience engagement and revisitation, which indicates their effectiveness in inducing ASMR. The visuals were removed to preserve only the auditory component, which was shortened to a 20-s audio clip. The same audio was shared across all conditions regardless of ASMR ability. By doing this, we examined if altering the auditory stimuli’s context could affect the emotional outcome of ASMR.

#### 2.2.3 ASMR videos

Eleven muted video stimuli were retrieved from YouTube based on their view count and congruence to the corresponding condition (fear, relaxation, and control) and auditory stimuli (tapping sounds). They were trimmed to 20 s to match the duration of the auditory stimuli. The five videos utilized for the relaxation condition were extracted from the original visuals where the ASMR audio was acquired (i.e., the visuals originated from the ASMR audio) to ensure the condition elicits the intended (positive) ASMR experience from the source material. In addition, five videos were used in the fear condition and picked based on their popularity, which we assumed indicated their capability to induce fear. These clips were obtained from horror movies and fearsome ASMR videos shared on YouTube. They were congruent to the ASMR tapping audio (e.g., raining in a forest at night, stalked by crawly monster steps, haunted house and sewer tour, and monster tapping closet; see Supplementary Appendix A) to ensure a congruent association between the audio and video. Finally, one emotionally neutral video clip was used in the control condition (a ball bouncing at a constant speed; see Supplementary Appendix A).

#### 2.2.4 ASMR checklist

Two questions needed to be answered before proceeding to the experiment to label participants as ASMR capable or incapable: (1) Have you ever experienced ASMR? (Yes/No); (2) Which type of ASMR are you capable of (select from Whispering, Chewing, Brushing, Tapping, Scratching, & Crinkling). Participants who selected “No” for the first question or did not choose “tapping” in the second question were allocated to the “ASMR Incapable” group.

#### 2.2.5 Self-assessment manikin scale

Self-assessment manikin scale (SAM) is a three-item pictorial assessment that measures arousal, valence, and dominance associated with an individual’s emotional reaction when reacting to various stimuli (Bradley and Lang, 1994). The current experiment adapted the 9-point version of the scale, removed the dominance scale, and replaced it with an original scale that accesses ASMR intensity (1 = “No arousal”/"No ASMR”/"Extremely Unpleasant” to 9 = “Extremely aroused”/"Extreme ASMR intensity”/"Extremely Pleasant”; see Supplementary Appendix B). This current assessment consisted of nine images for each scale, and each image of the scale was a visual representation of what each rating should feel like emotionally as a reference.

#### 2.2.6 Brief mood introspection scale

This scale is an open-source 4-point Likert mood scale that contains 16 mood adjectives (Mayer and Gaschke, 1988; Lively, Happy, Sad, Tired, Caring, Content, Gloomy, Jittery, Drowsy, Grouchy, Peppy, Nervous, Calm, Loving, Fed up, and Active). Participants had to report to what extent each adjective describes their current mood (1 = “Definitely do not feel,” 2 = “Do not feel,” 3 = “Slightly feel,” 4 = “Definitely Feel”). The data yielded were adjusted by reverse scoring components to reflect the pleasant–unpleasant mood or arousal–calm mood (Mayer and Cavallaro, 2019). Two BMIS were distributed; one was presented before the experiment, and the other was presented after the experiment. An overall pleasant–unpleasant or arousal–calm mood change score was obtained by deducting the BMIS before the experiment with the BMIS after the experiment. The reported Cronbach’s alpha for the questionnaire before and after the experiment were 0.84 and 0.87 for pleasant–unpleasant and 0.42 and 0.44 for arousal–calm, respectively.

### 2.3 Design

The current experiment used a 2 × 3 factorial design. Independent variables were the participant’s ASMR capability and the allocated priming conditions (i.e., control, fear, and relaxation). The dependent variables were (1) average SAM valence score, (2) average SAM arousal score, (3) average SAM ASMR intensity score, (4) BMIS unpleasant-pleasant mood change, and (5) BMIS arousal-calm mood change.

### 2.4 Procedure

#### 2.4.1 Pre-experiment phase

Participants were given a link to the web-based experimental program. Once consent was obtained, participants completed demographic information (age, gender, and nationality) and then completed a pre-screening questionnaire to identify as either ASMR capable or incapable. They were then allocated randomly to control, relaxation, or fear conditions and were given the BMIS to measure their mood before ASMR exposure.

#### 2.4.2 Experiment phase

Participants were then shown a muted video clip with the emotional context coherent to their grouping (i.e., neutral, relaxing, or fearsome). Once they watched the clip, participants were presented with a button to continue, which triggered a random audio clip (ASMR tapping) that lasted for another 20 s. Subsequently, participants were brought to four separate screens, where they had to provide their responses in the chronological order of (1) arousal, (2) valence, (3) ASMR intensity (see Supplementary Appendix B), and (4) interpretation of how the auditory stimulus is produced. After they completed SAM, participants were shown another muted video followed by ASMR audio, then SAM. This procedure was repeated five times; the combination of video and audio stimuli was randomly assorted for each trial and each run. All audio and video only appeared once throughout the entirety of the experiment. However, this is slightly different for the control conditions, as only one video was used as the neutral stimulus to serve as a consistent control; therefore, only the audio was randomized between trials and paired with said video.

#### 2.4.3 Post-experiment phase

**Figure 2:**
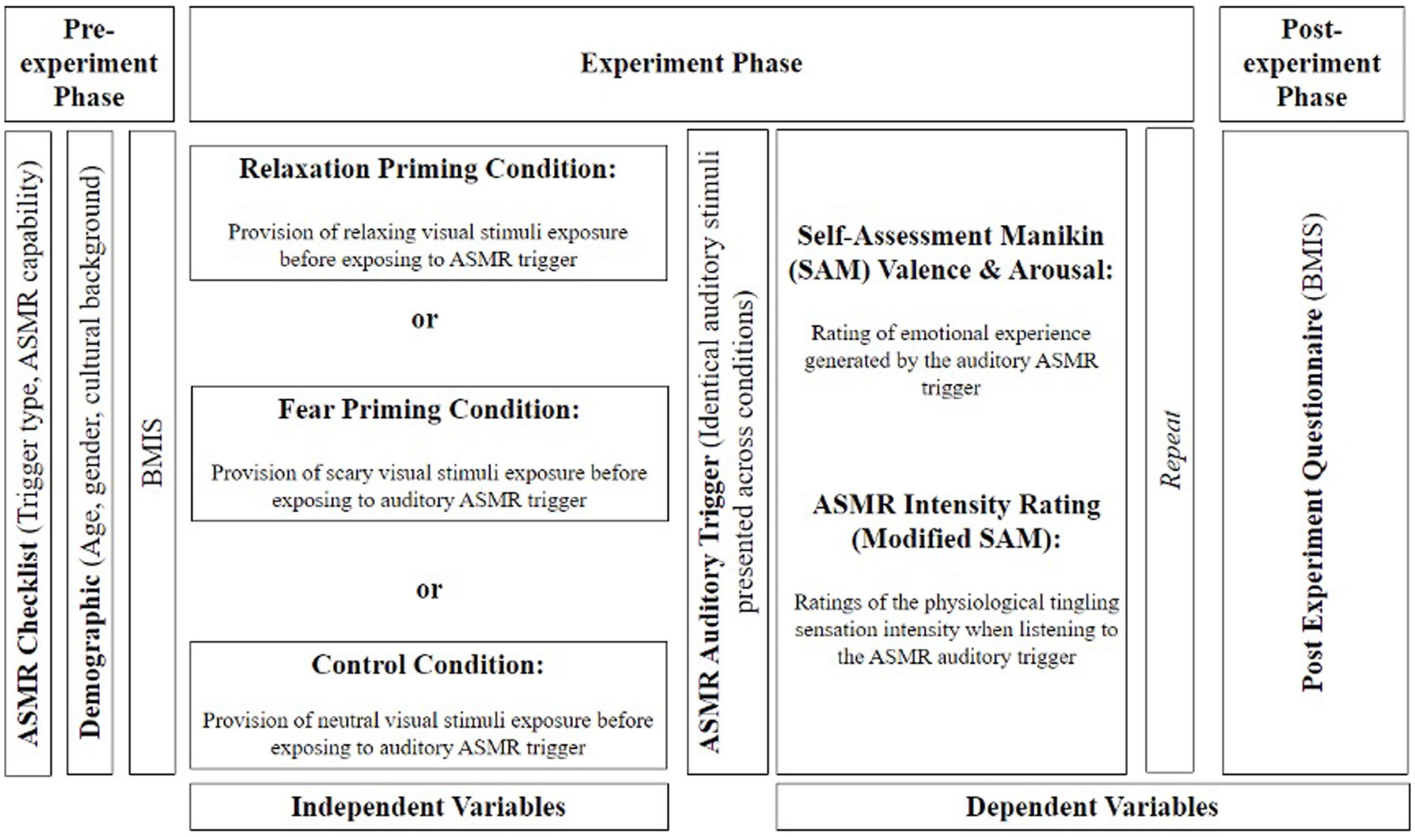
Experimental procedure.

When all five trials were completed, participants were instructed to complete another BMIS to measure their overall mood after the experiment, and participants were debriefed (see Figure 2).

### 2.5 Data analyses

Statistical analyses were carried out using the software IBM SPSS, version 27. The analysis was separated into three stages utilizing two-way ANOVA and one-way MANOVA.

#### 2.5.1 Analysis I

A 2 × 3 MANOVA with Bonferroni correction *post hoc* analysis is to be conducted to examine the overall differences in SAM valence, SAM arousal, SAM ASMR intensity, unpleasant–pleasant mood change, and arousal–calm mood change between ASMR capability and priming conditions. This would provide an overview of whether there will be significant main effects and interactions between the two independent variables (ASMR capability and emotional priming).

#### 2.5.2 Analysis II

Two one-way MANOVAs with Bonferroni correction post-hoc analysis are to be conducted to compare the same dependant variables, as the first analysis between priming conditions within ASMR capable and incapable conditions separately. Examining the differences in outcomes between priming conditions within the ASMR-incapable group allows for verification of whether our visual stimuli can induce the desired emotional outcome in the absence of ASMR. In contrast, examining the ASMR capable group allows examining how ASMR intensity varies between emotions for capable individuals testing the hypotheses: (1) ASMR is a neutral physiological response that is not exclusive to inducing contentment and (2) ASMR is an emotionally neutral physiological response that is not evoked by emotion.

#### 2.5.3 Analysis III

Three one-way ANOVA analyses will be conducted to compare dependent variables of the same priming condition between ASMR capability groups (e.g., capable fear group vs. incapable fear group). This allows examining the influence of ASMR on emotion and mood when ASMR is present compared to when it is absent. This enables us to test the hypotheses: (1) ASMR is a neutral physiological response that is not exclusive to inducing contentment, and (2) Fear-based ASMR would induce an opposite effect compared to relaxing ASMR, which increases arousal and decreases valence.

## 3 Results

Descriptive statistics for each group are presented in Table 1, which depicts the mean SAM arousal and valence score across all five trials, as well as unpleasant–pleasant mood change and arousal–calm mood change.

**Table 1:** Descriptive statistic of SAM and BMIS between ASMR capability and priming conditions.

Mean (SD)						
	ASMR Incapable (*n* = 51)			ASMR Capable (*n* = 129)		
	Control (*n* = 16)	Fear (*n* = 16)	Relaxation (*n* = 19)	Control (*n* = 50)	Fear (*n* = 33)	Relaxation (*n* = 46)
Self-assessment manikin (SAM)						
Arousal	3.13 (1.23)	4.25 (2.00)	2.78 (1.02)	3.16 (1.45)	4.58 (1.50)	3.33 (1.51)
Valence	4.83 (0.86)	4.71 (0.74)	5.41 (1.06)	5.82 (1.15)	5.10 (1.41)	6.20 (0.96)
ASMR intensity	3.51 (1.58)	3.54 (1.80)	3.01 (1.69)	4.12 (1.65)	4.52 (2.12)	4.73 (1.80)
The brief mood introspection scale (BMIS)						
Unpleasant-pleasant mood change	−1.25 (5.03)	−5.00 (6.31)	1.16 (3.20)	1.60 (4.76)	−3.45 (7.40)	2.13 (4.63)
Arousal-calm mood change	−1.25 (3.00)	1.31 (2.89)	−2.00 (2.52)	−1.04 (3.08)	0.45 (4.09)	−1.72 (2.44)

SD, standard deviation; BMIS scores are obtained by deducting pre-experiment BMIS scores with post-experiment BMIS score; The lower the unpleasant–pleasant mood change score, the more unpleasant the mood; The lower the arousal–calm mood change score, the calmer the mood.

A total of 180 data points were analyzed; 51 participants were labeled as incapable of experiencing ASMR (28.3%) and 129 participants were reported to be capable of experiencing ASMR tapping (71.7%). The ASMR incapable group consisted of 16 participants in the control group (31.4%), 16 in the fear condition (31.4%), and 19 in the relaxation condition (37.3%), while the ASMR capable group consisted of 50 participants in the control condition (38.8%), 33 in the fear condition (25.6%), and 46 in the relaxation condition (35.7%). No extreme outliers were identified.

**Figure 3:**
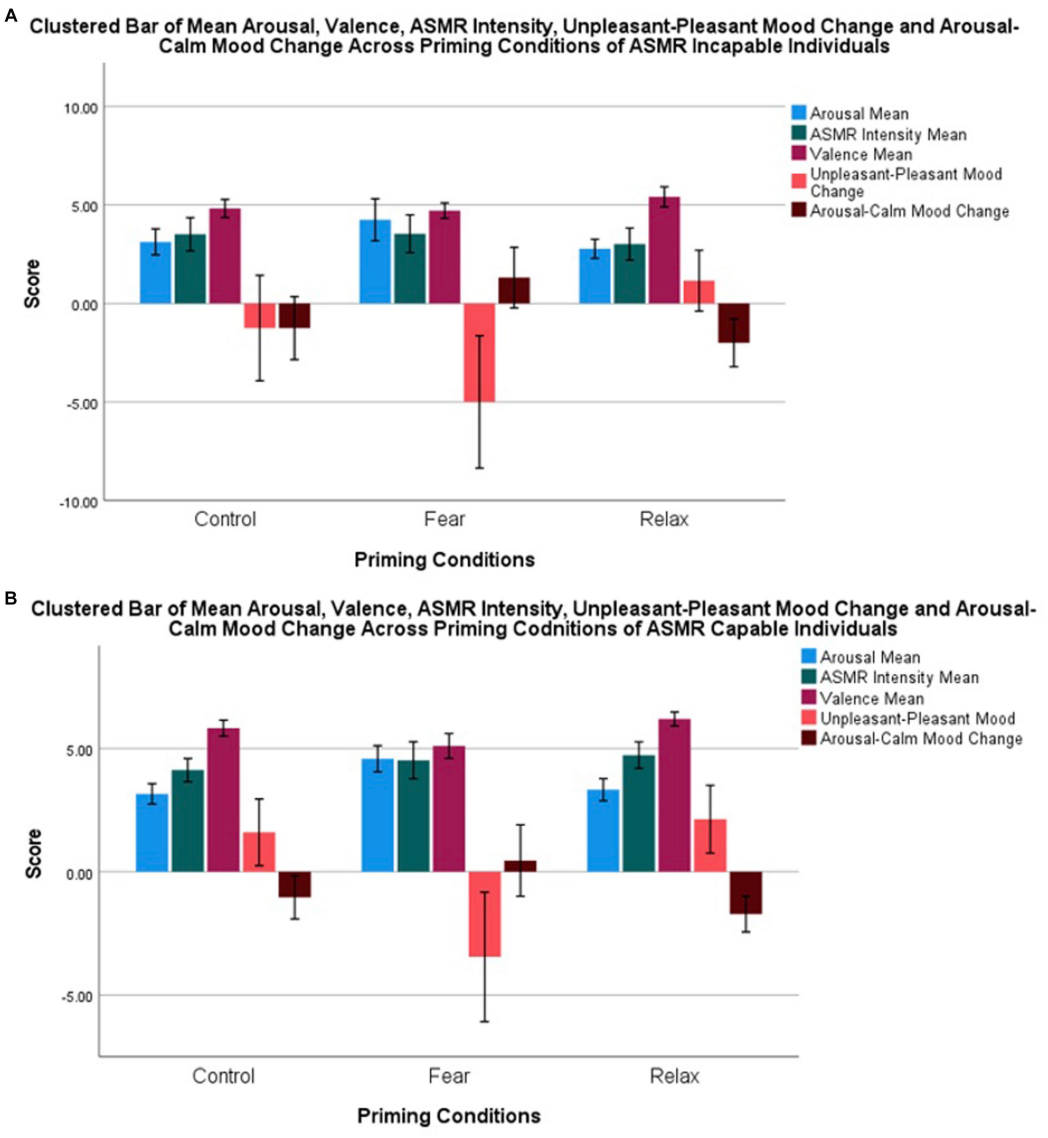
Cluster bar of mean arousal, valence, ASMR intensity, unpleasant–pleasant mood change, and arousal–calm mood changes of ASMR-incapable **(A)** and ASMR-capable individuals **(B)**.

The average SAM arousal score, average SAM ASMR intensity score, average SAM valence score, BMIS unpleasant-pleasant mood change, BMIS, and arousal-calm mood change between ASMR capability group are graphically displayed as a cluster bar chart (see Figure 3).

### 3.1 Analysis I—main effect and interaction

A 2 × 3 MANOVA analysis was conducted to compare dependent variables (DV) between ASMR capability and priming conditions at a 95% confidence interval. This analysis highlighted that there are significant results within the main effect of ASMR capability [*F*(5, 170) = 5.82, *p* < 0.001; Wilk’s Λ = 0.854, η^2^ = 0.146], whereas univariate analysis suggested there are significant differences between ASMR intensity [*F*(1, 174) = 13.64, *p* < 0.001, η^2^ = 0.073] and valence [*F*(1, 174) = 15.73, *p* < 0.001, η^2^ = 0.083]. However, there was no significant differences for arousal [*F*(1, 174) = 1.54, *p* = 0.22, η^2^ = 0.009] and arousal–calm mood change [*F*(1, 174) = 0.06, *p* = 0.81, η^2^ < 0.001]. Although unpleasant–pleasant mood change was significant within the main effect, it showed a significant effect in Levene’s test of equality of error variances, which indicated unequal variances [*F*(5, 174) = 4.12, *p* < 0.05]. Thus, Welch ANOVA will be conducted.

Moreover, a significant main effect was also found in priming conditions [*F*(10, 340) = 6.36, *p* < 0.001; Wilk’s Λ = 0.710, η^2^ = 0.157]. However, univariate analyses suggested that arousal [*F*(2, 174) = 12.24, p < 0.001, η^2^ = 0.123], valence [*F*(2, 174) = 8.05, *p* < 0.05, η^2^ = 0.085], and arousal–calm mood change [*F*(2, 174) = 9.97, *p* < 0.001, η^2^ = 0.103] were significantly different between priming conditions except for ASMR intensity [*F*(2, 174) = 0.17, *p* = 0.85, η^2^ = 0.002]. Regarding the interaction between ASMR capability and priming condition, there were no significant multivariate effect [*F*(10, 340) = 0.66, *p* = 0.76; Wilk’s Λ = 0.962, η^2^ = 0.019] nor differences in arousal [*F*(2, 174) = 0.39, *p* = 0.68, η^2^ = 0.004], valence [*F*(2, 174) = 0.90, *p* = 0.41, η^2^ = 0.010], ASMR intensity [*F*(2, 174) = 1.27, *p* = 0.29, η^2^ = 0.014], and arousal–calm mood [*F*(2, 174) = 0.49, *p* = 0.61, η^2^ = 0.006; see Table 2].

**Table 2:** The 2 × 3 MANOVA analysis on main effect and interaction and Welch ANOVA on unpleasant–pleasant mood changes.

	Wilk’s Λ	*df*	*df*(Error)	*F*	*p*	η^2^
ASMR capability	0.854	5	170	5.82	<0.001	0.146
ASMR intensity		1	174	13.64	<0.001	0.073
Valence		1	174	15.73	<0.001	0.083
Arousal		1	174	1.54	0.22	0.009
Arousal–calm mood change		1	174	0.06	0.81	<0.001
Priming condition	0.710	10	340	6.36	<0.001	0.157
ASMR intensity		2	174	0.17	0.85	0.002
Valence		2	174	8.05	<0.05	0.085
Arousal		2	174	12.24	<0.001	0.123
Arousal–calm mood change		2	174	9.97	<0.001	0.103
ASMR capability × priming condition	0.962	10	340	0.66	0.76	0.019
ASMR intensity		2	174	0.39	0.68	0.004
Valence		2	174	0.90	0.41	0.010
Arousal		2	174	1.27	0.29	0.014
Arousal–calm mood change		2	174	0.49	0.61	0.006
Unpleasant–pleasant mood change		1	99.27	4.77	<0.05	

Post-hoc analysis on the main effect of priming conditions using Bonferroni correction tests showed that there was no significant difference in any of the dependent variables between the control and relaxation conditions. In contrast, all dependent variables except for ASMR intensity were significantly different when comparing fear against the control and relaxation conditions. Both control and relaxation had significantly higher (positive) valence, lesser arousal, lower arousal–calm mood, and higher pleasant–unpleasant mood change compared to fear (see Table 1). A lower arousal–calm mood change value refers to a calmer mood, and a higher pleasant–unpleasant mood change value refers to a more pleasant mood. There were no significant differences in ASMR intensity between all three priming conditions. Contrasts between ASMR capability, arousal, and arousal–calm mood change were not significantly different between ASMR capable and incapable samples. However, there were significantly higher levels of ASMR intensity and positive valence within the ASMR capable group than the incapable group (see Table 1).

Welch ANOVA was conducted for unpleasant–pleasant mood change due to unequal variance. This demonstrated that the ASMR capable group had significantly higher unpleasant–pleasant mood changes compared to the ASMR incapable group at *F*(1, 99.27) = 4.77, *p* < 0.05. This indicates that the ASMR capable group had a significant increase in pleasantness in mood after the experiment. Furthermore, a significant main effect of the priming condition was also found from Welch’s statistics at *F*(2,103.97), *p* < 0.001. Bonferroni post-hoc test suggested no significant differences in unpleasant–pleasant mood change between control (*M* = 0.91, *SD* = 4.94) and relaxation conditions (*M* = 1.85, *SD* = 4.26). However, the fear condition demonstrated a significantly more unpleasant mood when compared to control and relaxation (*M* = −3.45, *SD* = 7.03; see Table 2).

### 3.2 Analysis II—ASMR capability specific between priming conditions analysis

Two one-way MANOVAs were conducted to examine how priming conditions within ASMR-capable or incapable groups differ from the other priming conditions within the same group. For the ASMR-incapable group, there were significant multivariate effects at *F*(10, 88) = 2.56, *p* < 0.05; Wilk’s Λ = 0.601, η^2^ = 0.225, and univariate analysis suggested significant differences for arousal–clam mood changes [*F*(2, 48) = 6.52, *p* < 0.01, η^2^ = 0.214] and unpleasant–pleasant mood changes [*F*(2, 48) = 6.84, *p* < 0.01, η^2^ = 0.214], and borderline significant difference with valence [*F*(2, 48) = 3.04, *p* = 0.057, η^2^ = 0.562]. However, ASMR intensity demonstrated a non-significant difference at *F*(2, 48) = 0.55, *p* = 0.58, η^2^ = 0.022. Furthermore, arousal scores were found significant in Levene’s statistics at *F*(2, 48) = 4.30, *p* < 0.05. This indicates unequal variances, and Welsch’s ANOVA was conducted, which resulted in significant differences at *F*(2, 28.57) = 3.52, *p* < 0.05. Bonferroni *post-hoc* tests suggested that there was no significant difference in arousal, arousal–calm mood change, and unpleasant–pleasant mood change between control and relaxation conditions. However, the fear condition had significantly higher arousal, aroused mood, and unpleasant mood (see Table 1) when compared to the relaxation condition. Furthermore, no significant differences were found in ASMR intensity and valence among all three conditions (see Table 3).

**Table 3:** ASMR capability specific between priming conditions MANOVA analysis.

	Wilk’s Λ	*df*	*df*(Error)	*F*	*p*	η^2^
ASMR incapable	0.601	10	88	2.56	<0.05	0.225
ASMR intensity		2	48	0.55	0.58	0.022
Valence		2	48	3.04	0.057	0.562
Arousal		2	28.57	3.52	<0.05	
Arousal–calm mood change		2	48	6.52	<0.01	0.214
Unpleasant–pleasant mood change		2	48	6.84	<0.01	0.214
ASMR capable	0.657	10	244	5.71	<0.001	0.190
ASMR intensity		2	126	1.35	0.26	0.190
Valence		2	126	8.59	<0.001	0.120
Arousal		2	126	10.23	<0.001	0.140
Arousal–calm mood change		2	71.06	7.57	<0.001	
Unpleasant–pleasant mood change		2	71.44	3.77	<0.05	

With regard to the ASMR capable group, arousal–calm mood change [*F*(2, 126) = 4.04, *p* < 0.05] and unpleasant–pleasant mood change [*F*(2, 126) = 6.91, *p* < 0.01] were significant in the Levene statistics. Thus, Welch ANOVA tests were carried out where unpleasant–pleasant mood change was significant at *F*(2, 71.06) = 7.57, *p* < 0.01, and arousal–calm mood changes were significant at *F*(2, 71.44) = 3.77, *p* < 0.05. Significant multivariate effect was reported at *F*(10, 244) = 5.71, *p* < 0.001; Wilk’s Λ = 0.657, η^2^ = 0.190. Arousal and valence were found significant at *F*(2, 126) = 10.23, *p* < 0.001, η^2^ = 0.140 and *F*(2, 126) = 8.59, *p* < 0.001, η^2^ = 0.120. Bonferroni post-hoc test showed no significant difference between control and relaxation conditions for all dependent variables. However, fear conditions demonstrated significantly higher arousal, negative valence, aroused mood, and unpleasant mood compared to control and relaxation, except for ASMR intensity (see Table 1), where all conditions demonstrated no significant differences (see Table 3).

### 3.3 Analysis III—priming conditions specific between ASMR capability analysis

Three one-way MANOVAs were conducted to compare how the same priming condition differs between ASMR capability groups. First, control conditions demonstrated a significant multivariate effect at *F*(5, 60) = 0.793, *p* < 0.05; Wilk’s Λ = 0.793, η^2^ = 0.207, and significant increased positive valence and pleasantness in the mood when ASMR is present at *F*(1, 64) = 10.24, *p* < 0.01, η^2^ = 0.138 and *F*(1, 64) = 4.23, *p* < 0.05, η^2^ = 0.062. However, no significant differences were identified for arousal [*F*(1, 64) < 0.01, *p* = 0.93, η^2^ < 0.001], ASMR intensity [*F*(1, 64) = 1.69, *p* = 0.20, η^2^ = 0.026], and unpleasant–pleasant mood change [*F*(1, 64) = 0.06, *p* = 0.81, η^2^ = 0.001]. Second, the fear condition did not show a significant multivariate effect [*F*(5, 43) = 0.85, *p* = 0.52; Wilk’s Λ = 0.910, η^2^ = 0.090] nor results for all dependent variables when compared between ASMR-capable and incapable samples. However, arousal is at *F*(1, 47) = 0.42, *p* = 0.52, η^2^ = 0.009, valence at *F*(1, 47) = 1.07, *p* = 0.31, η^2^ = 0.022, ASMR intensity at *F*(1, 47) = 2.55, *p* = 0.18, η^2^ = 0.051, unpleasant–pleasant mood change at *F*(1, 47) = 0.52, *p* = 0.48, η^2^ = 0.011, and arousal–calm mood change at *F*(1, 47) = 0.57, *p* = 0.46, η^2^ = 0.012. Finally, the relaxation condition showed significant multivariate effect at *F*(5, 59) = 3.29, p < 0.05; Wilk’s Λ = 0.782, η^2^ = 0.218 and demonstrated that ASMR intensity and valence were significant at *F*(1, 63) = 12.65, *p* < 0.001, η^2^ = 0.167 and *F*(1, 63) = 8.29, *p* < 0.01, η^2^ = 0.118, respectively. However, there were no significant results for arousal [*F*(1, 63) = 2.10, *p* = 0.15, η^2^ = 0.032], unpleasant–pleasant mood change [*F*(1, 63) = 0.70, *p* = 0.41, η^2^ = 0.011], and arousal–calm mood change [*F*(1, 63) = 0.18, *p* = 0.68, η^2^ = 0.032]. Furthermore, arousal demonstrated significant Levene’s statistics at *F*(1, 63) = 5.59, *p* < 0.05, indicating unequal variances. Regardless, Welch ANOVA showed a non-significant result for arousal at *F*(1, 49.77) = 2.91, *p* = 0.094 (see Table 4).

**Table 4:** Priming conditions specific between ASMR capability MANOVA analysis.

	Wilk’s Λ	*df*	*df* (Error)	*F*	*p*	η^2^
Control	0.793	5	60	0.793	<0.05	0.207
ASMR intensity		1	64	1.69	0.20	0.026
Valence		1	64	10.24	<0.01	0.138
Arousal		1	64	<0.01	0.93	<0.001
Arousal–calm mood change		1	64	0.06	0.81	0.001
Unpleasant–pleasant mood change		1	64	4.23	<0.05	0.062
Fear	0.910	5	43	0.85	0.52	0.090
ASMR intensity		1	47	2.55	0.18	0.051
Valence		1	47	1.07	0.31	0.022
Arousal		1	47	0.42	0.52	0.009
Arousal–calm mood change		1	47	0.57	0.46	0.012
Unpleasant–pleasant mood change		1	47	0.52	0.48	0.011
Relaxation	0.782	5	59	32.29	<0.05	0.218
ASMR intensity		1	63	12.65	<0.001	0.167
Valence		1	63	8.29	<0.01	0.032
Arousal		1	49.77	2.91	0.09	
Arousal–calm mood change		1	63	0.70	0.41	0.011
Unpleasant–pleasant mood change		1	63	0.18	0.68	0.032

## 4 Discussion

This study employs popular YouTube videos to test the hypothesis that positive emotions are not solely determined by the ASMR sensation itself but rather by the sound that amplifies emotions triggered by visual stimuli, whether positive or negative. We demonstrate that the same ASMR tapping sound, when primed with videos of different emotional valences (fear, relaxation, and control), elicits an amplification effect on subjects, particularly those who are ASMR-capable.

As predicted, fear-based ASMR increased arousal and decreased positive valence compared to relaxation-based ASMR. Although the role of ASMR in a fearful context might require further testing, the results demonstrated that ASMR occurs also under non-relaxed experience. The sensation itself is not responsible for determining the emotional outcome, but it could possibly serve as a physical amplifier of the emotion appraised. If ASMR was a response that was always accompanied by relaxation, fear priming should not have had the effect of reducing positive valence and increasing arousal. The level of ASMR intensity does not significantly differ across priming conditions. This indicates that the difference in emotional outcome is not caused by the absence of ASMR tingling sensation but rather by the emotion perceived in the context.

Meanwhile, both control and relaxation conditions demonstrated significant increases in valence for ASMR-capable individuals, which aligns with our assumption that ASMR acts as a medium that physically amplifies pre-existing emotions since individuals who are ASMR-capable demonstrated the same emotional score direction as ASMR-incapable individuals but to a greater extent.

The study adds its findings to the debate of whether ASMR is, in fact, the same phenomenon as frisson. In the literature, we have seen that it is inherently difficult to distinguish the physiological sensations between ASMR and frisson, with a sensation of contentment being the only differentiator highlighted so far. Thus, being able to feel ASMR in both positive and negative emotional contexts supports the idea that the two might be descriptions of the same phenomenon.

### 4.1 Alternative interpretations, limitations, and future directions

There is a non-significant difference between control and relaxation priming conditions for ASMR-capable individuals, which could also be evidence of bias in the ASMR community. As Smith and Snider (2019) showcased the community’s encouragement toward the view of ASMR as a relaxation-based sensation, this mainstream impression of ASMR would ultimately provide enough context needed to determine the initial emotion. Subsequently, the capable control group may have decided to anticipate ASMR as a relaxing response, which amplifies their contentment. This could explain the non-significant differences between control and relaxation conditions.

One could interpret the effect induced by fear-based ASMR as suppression of contentment induction when interpreting stimuli negatively. To elaborate further, while the relaxation condition demonstrated that the presence of ASMR leads to improved valence, this effect is absent in the fear condition as none of the dependent variables have shown to be significantly different between the capable and incapable groups. This valence-improving effect in the relaxation condition seems suppressed under a fear-related context. If this is the case, the result would indicate that there is a certain threshold before ASMR becomes ineffective in inducing contentment, which may be an exciting aspect for future investigations wanting to explore the parameters of ASMR as a therapeutic or meditative medium.

Although the results might favor the suppression theory since there were no significant differences in emotions across ASMR capability groups in the fear condition, it is possible that the amplification hypothesis still stands due to a response bias. This assumes that most ASMR-incapable individuals do not know what ASMR-tapping feels like and resort to central tendency bias to avoid giving extreme responses, which causes the non-significant statistics between fear-incapable and capable groups. This is supported by no significant differences in ASMR intensity for the fear priming conditions between ASMR capability groups. Similarly, ASMR intensity does not significantly differ between capability groups for the control condition. We can assume that the problem does not arise from the ASMR capable group since both priming conditions shared the same level of ASMR intensity with the capable relaxation group, which was statistically different than its incapable counterparts. Overall, some non-significant ASMR intensity differences found between the capabilities group (i.e., fear and control conditions) and the average ASMR intensity score in the incapable group being over three points are assumed to be caused by response bias. Regardless, the main effect of ASMR capability on ASMR intensity score remains statistically significant, which indicates that the capability groups are sufficiently different for the analyses.

To confirm and eliminate our suspicion of response bias at play, future research could adapt new baselines with ASMR-capable individuals who would go through the same priming procedure but do not receive auditory ASMR stimulation to eliminate the possibility of experiencing ASMR as part of the ASMR-incapable group (control group). Future incorporation of personality traits measurements may also be useful for sanity checking for grouping errors between ASMR capability groups, as ASMR-capable individuals have shown to have significantly higher scores on openness-to-experience and neuroticism but significantly lower levels of conscientiousness, extraversion, and agreeableness (e.g., Fredborg et al., 2017; McErlean and Banissy, 2017).

Alternatively, more direct measurements could be implemented to investigate fear-related ASMR to counteract questionnaire biases. For instance, fMRI (Smith et al., 2019) or EEG (Fredborg et al., 2021) was used to examine areas such as the right cingulate gyrus, right paracentral lobule, and bilateral thalamus that indicate ASMR experience to ensure appropriate and more precise groupings of ASMR-capable and incapable individuals for better clarity of the true effect with fear-related ASMR when comparing affective differences across capability groups.

Furthermore, measurement errors caused by equipment differences among participants may be present. Since the investigation was conducted online, we had no control over the monitor size or auditory apparatus utilized, and participants’ different monitors or headphones might have influenced their perception of the auditory stimuli or priming material, which in turn may have influenced their emotional ratings. Regarding the perception of auditory stimuli, Koumura et al. (2020) have suggested that the intensity of the frisson-like sensation from ASMR is linked to the acoustic features of the auditory stimuli, such as amplitude, spectral centroid, and spectral bandwidth. The lack of control in audio devices could influence the perceptibility of acoustic components; subsequently, failing to perceive acoustic intricacies could disable the initiation of the ASMR experience. However, significantly higher ASMR intensity for capable individuals would indicate the presence of ASMR, which further implies that participants were able to perceive our auditory material appropriately and that the effect of equipment variance did not significantly impact the results.

Future research could objectively investigate whether ASMR is a susceptibility in physiological response that causes one to be extra sensitive toward certain auditory stimuli, which is independent of emotions. This is not unlike those who are more sensitive to skin contact and are labeled as “ticklish.” Translating this into the context of ASMR, ASMR-capable individuals may simply be more sensitive to a certain pitch, sound patterns, tone, or other auditory properties of the ASMR trigger, which leads to a physical response to the extra sensitivity.

## 5 Conclusion

In conclusion, ASMR was shown to be present under different emotional states, suggesting that the effect of ASMR can be altered by influencing the affective interpretation of ASMR stimuli, supporting the idea that, similar to frisson, it is a physically amplified, overwhelming emotional experience. With relaxation-based ASMR, the tingling sensations may serve as an amplifier to boost the pre-existing contentment. In contrast, fear-based ASMR seems to suppress contentment induction when the stimuli are interpreted negatively, and significantly higher arousal and negative valence are induced by attaching a fearful context to ASMR stimuli, showing that ASMR does not always induce relaxation.

## Data availability statement

The raw data supporting the conclusions of this article will be made available by the authors, without undue reservation.

## Ethics statement

The studies involving human participants were reviewed and approved by the University College London, Department of Information Study Ethics Chair. The participants provided their informed consent to participate in this study online. The studies were conducted in accordance with the local legislation and institutional requirements.

## Author contributions

WLL and DR: writing, review and editing, data curation, formal analysis, and investigation. WLL: data collection and literature review. DR: research supervision. All authors contributed to the article and approved the submitted version.

## Footnotes

^1^ https://www.reddit.com/r/asmr/
