# Supplementary material for: Autonomous sensory Meridian response as a physically felt signature of positive and negative emotions
Source: Front Psychol. 2024 Mar 1;15:1183996. doi: 10.3389/fpsyg.2024.1183996 (PMC10959090; doi:10.3389/fpsyg.2024.1183996)
Supplement: Supplementary file 1 [file Data_sheet.zip › fpsyg.2024.1183996 -Datasheet/Data Sheet 1.docx]

# Appendix A

Relax Video #1 ([https://www.youtube.com/watch?v=sIgkTYTWPz8](about:blank)) [37:45 - 38:05],

Relax Video #2 ([https://www.youtube.com/watch?v=kfuyuZ0zP-8](about:blank)) [2:40 - 3:00],

Relax Video #3 ([https://www.youtube.com/watch?v=cXIXM2rjqM8](about:blank)) [4:55 - 5:15],

Relax Video #4 ([https://www.youtube.com/watch?v=sQ9k-FVz7yI](about:blank)) [5:30 - 5:50],

Relax Video #5 ([https://www.youtube.com/watch?v=-q4jFTOpKpc](about:blank)) [16:36 - 16:56],

Control Video ([https://www.youtube.com/watch?v=zsdPYFPTdw0](about:blank)) [0:00 - 0:20],

Fear Video #1 ([https://www.youtube.com/watch?v=tT6hjeRpJq8](about:blank)) [1:20 - 1:40],

Fear Video #2 ([https://www.youtube.com/watch?v=fnyPuH5LFbI](about:blank)) [13:10 - 13:30],

Fear Video #3 ([https://www.youtube.com/watch?v=cmYsRcLMvO8](about:blank)) [1:00 - 1:20],

Fear Video #4 ([https://www.youtube.com/watch?v=zHZxmkzdlmQ](about:blank)) [10:25 - 10:45],

Fear Video #5 ([https://www.youtube.com/watch?v=hqsQY-QXI2U](about:blank)) [20:05 - 20:25].

# Appendix B


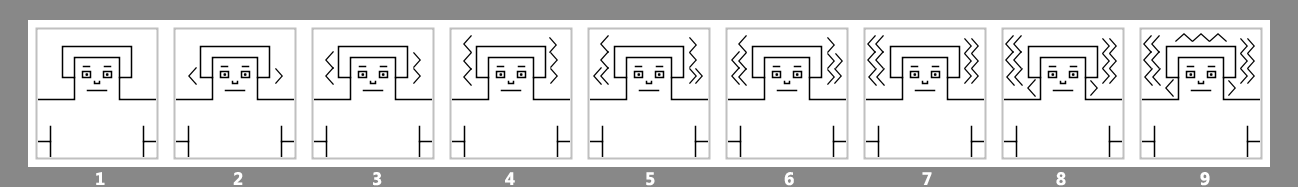


*Figure 1.* New SAM ASMR intensity scale (1 = No ASMR, 9 = Extreme ASMR intensity)
